# Parturients Vulnerability to Hyperbaric Bupivacaine-Induced Neurotoxicity After Intrathecal Administration: A Series of Four Cases

**DOI:** 10.7759/cureus.106179

**Published:** 2026-03-31

**Authors:** Kenya Gupta, Jyoti Kanwat, Gopal Jalwal, Swati Das

**Affiliations:** 1 Anaesthesiology, All India Institute of Medical Sciences (AIIMS) Bathinda, Bathinda, IND; 2 Anaesthesiology and Critical Sciences, All India Institute of Medical Sciences (AIIMS) Bathinda, Bathinda, IND

**Keywords:** case series, hyperbaric bupivacaine, neurotoxicity, parturients, pregnancy vulnerability, spinal anaesthesia

## Abstract

Spinal anaesthesia using intrathecal hyperbaric bupivacaine is widely employed in caesarean sections due to its rapid onset, effective analgesia, and minimal foetal transfer. While generally safe, neurotoxicity following intrathecal bupivacaine is rare. This case series highlights an unusual cluster of neurotoxic events observed exclusively in pregnant patients. Four parturients undergoing lower segment caesarean section (LSCS) under spinal anaesthesia with 10 mg (2 ml) of 0.5% hyperbaric bupivacaine developed neurological complications. Neurological symptoms ranging from headache, nausea, vomiting, altered sensorium to generalized tonic-clonic seizures within two to three hours postoperatively. Two of the parturients required mechanical ventilation due to recurrent seizures and cerebral edema evident on MRI. Supportive management with airway protection, benzodiazepines, anti-edema therapy, and antiepileptics led to full recovery without long-term sequelae. Importantly, no neurotoxicity was reported in non-pregnant patients who were exposed to the same drug batch. The findings suggest a pregnancy-specific vulnerability to intrathecal bupivacaine neurotoxicity. Physiological changes in pregnancy-such as reduced cerebrospinal fluid volume, venous engorgement, hormonal modulation of neuronal excitability, and altered blood-neural barrier permeability-may predispose parturients to enhanced CNS exposure and toxicity, even at standard doses. The clustering of cases linked to a single suspected faulty batch further underscores the need for stringent pharmacovigilance and drug quality assurance. Early recognition, airway management, and supportive therapy are crucial for desired outcomes. Enhanced vigilance, dose optimization, and robust quality control of anaesthetic agents are essential to improve obstetric anaesthesia safety.

## Introduction

Spinal anaesthesia using intrathecal hyperbaric bupivacaine is widely accepted as the preferred technique for caesarean delivery, offering rapid onset and reliable surgical anaesthesia [1]. Its use has been extensively studied in obstetric practice, demonstrating dense motor block and consistent sensory levels suitable for caesarean section [2]. These advantages, combined with a favourable safety profile in routine obstetric settings, support its continued use in both elective and emergency scenarios [3]. Pharmacologically, bupivacaine provides a long duration of action and stable haemodynamic characteristics when used appropriately, contributing to its favourable clinical performance [4]. Contemporary observational data further support its effectiveness and widespread use in caesarean section anaesthesia [5].

Despite this favourable profile, bupivacaine is not completely without risk. Several clinical case reports have documented rare but severe neurological complications, including seizures and status epilepticus, following intrathecal administration [6,7]. Experimental evidence has also demonstrated neurotoxic potential under certain conditions, suggesting that susceptibility may depend on dose, concentration, or patient-specific factors [8]. Additional clinical observations describe transient or persistent neurological deficits following spinal anaesthesia, reinforcing the need for vigilance when administering intrathecal local anaesthetics [9,10].

The mechanisms underlying neurotoxicity are multifactorial. Experimental studies implicate mitochondrial injury, apoptosis pathways, and local inflammatory changes in the spinal cord [11]. Pregnancy-related anatomical and physiological changes, such as reduced cerebrospinal fluid volume, engorged epidural veins, and increased intra-abdominal pressure, may enhance cephalad spread of intrathecal solutions, further influencing clinical response and potential toxicity [1-3].

In this context, we report a case series of four postpartum patients who developed acute neurological symptoms following intrathecal administration of 10 mg hyperbaric bupivacaine for caesarean delivery. Interestingly, at the same time, other non-pregnant patients who received the injection of bupivacaine in spinal anaesthesia for other surgeries with the same batch did not exhibit any neurological side effects. All parturients gave a negative history for chronic hypertension, pre-eclampsia, eclampsia, seizure disorder, or infections like meningitis, encephalitis, or any other metabolic imbalances.

## Case presentation

Case 1

**Figure 1:**
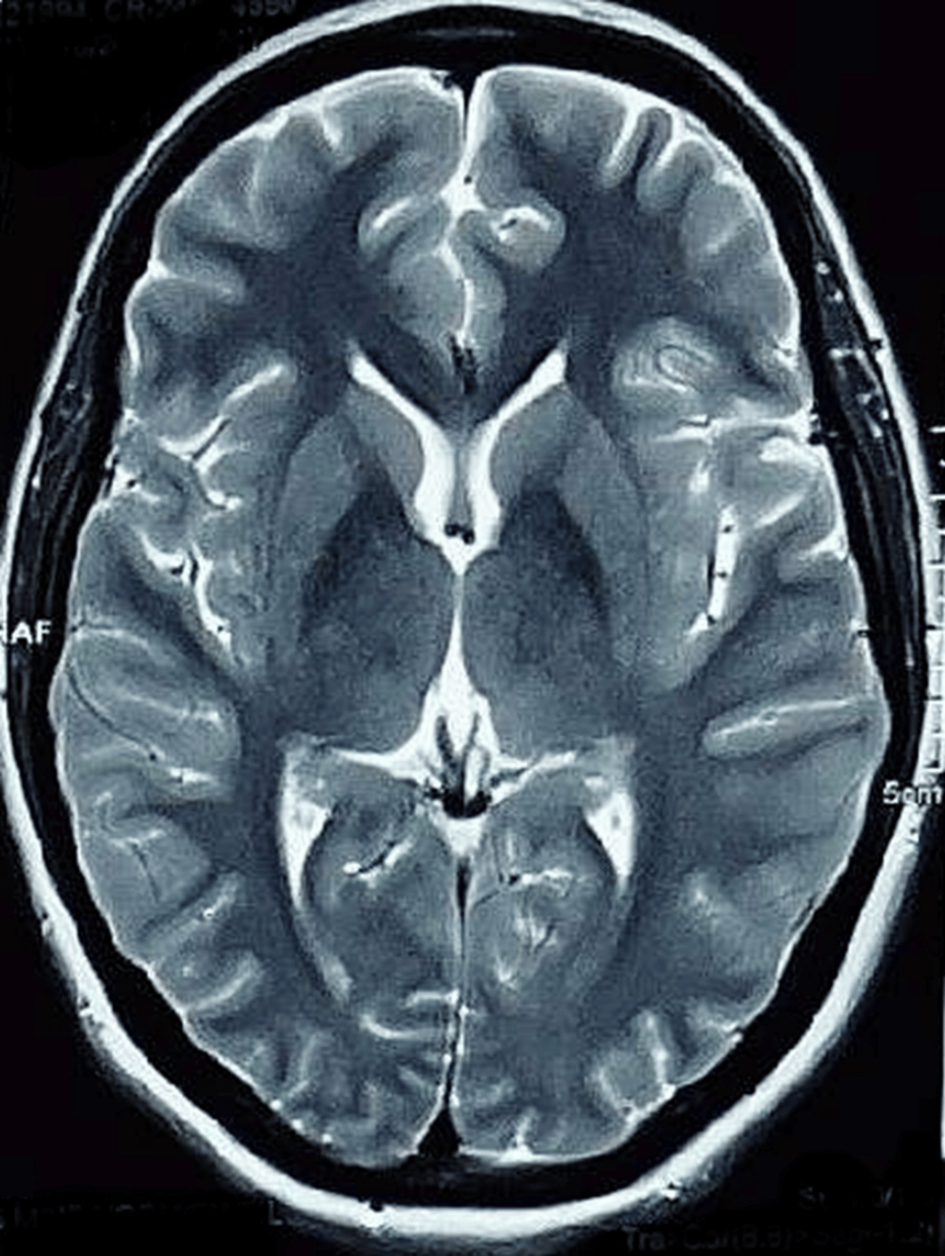
T2-weighed MRI brain sequence with normal results

A 26-year-old female, P2L2, underwent an emergency lower segment caesarean section (LSCS) at term due to scar tenderness of a previous cesarean section. Anaesthesia was administered via subarachnoid block using 10 mg of 0.5% hyperbaric bupivacaine. The intraoperative and immediate postpartum period were uneventful; however, approximately two hours later, she developed a headache and nausea, followed by two episodes of vomiting. This was soon complicated by two generalized tonic-clonic seizures (GTCS), each lasting 3 to 5 minutes. Clinical examination revealed no neck rigidity, but her responsiveness was decreased with a Glasgow Coma Scale (GCS) of E2V2M4. She was intubated and shifted to the ICU for further management, where she was put on mechanical ventilation, antiepileptics, and mannitol. T2-weighted MRI showed normal findings (Figure 1). Slowly, with supportive treatment, her GCS improved, and she was extubated after two days without any neurological sequelae.

Case 2

A 21-year-old female, P2L2, underwent an elective LSCS under subarachnoid block with 2 ml of 0.5% hyperbaric bupivacaine. Approximately three hours postoperatively, she began complaining of a headache, followed by two to three episodes of vomiting. She subsequently exhibited altered behavior and irritability, along with irrelevant speech, which persisted for about three hours. No further neurological signs or symptoms were noted. The patient remained hemodynamically stable, was kept under close observation for 48 hours, and was subsequently discharged. On follow-up after one week, the patient reported no new complaints.

Case 3

A 28-year-old female, P1L1, underwent an emergency LSCS for cephalopelvic disproportion (CPD) under subarachnoid block with 2 ml of 0.5% hyperbaric bupivacaine. Two hours after the procedure, she developed a headache followed by abnormal body movements and a GTCS. Her GCS was E3V2M5. Supportive management was initiated. Her GCS scores showed gradual improvement over 72 hours, during which she remained hemodynamically stable and was able to tolerate oral feeds. The patient was discharged subsequently.

Case 4

**Figure 2:**
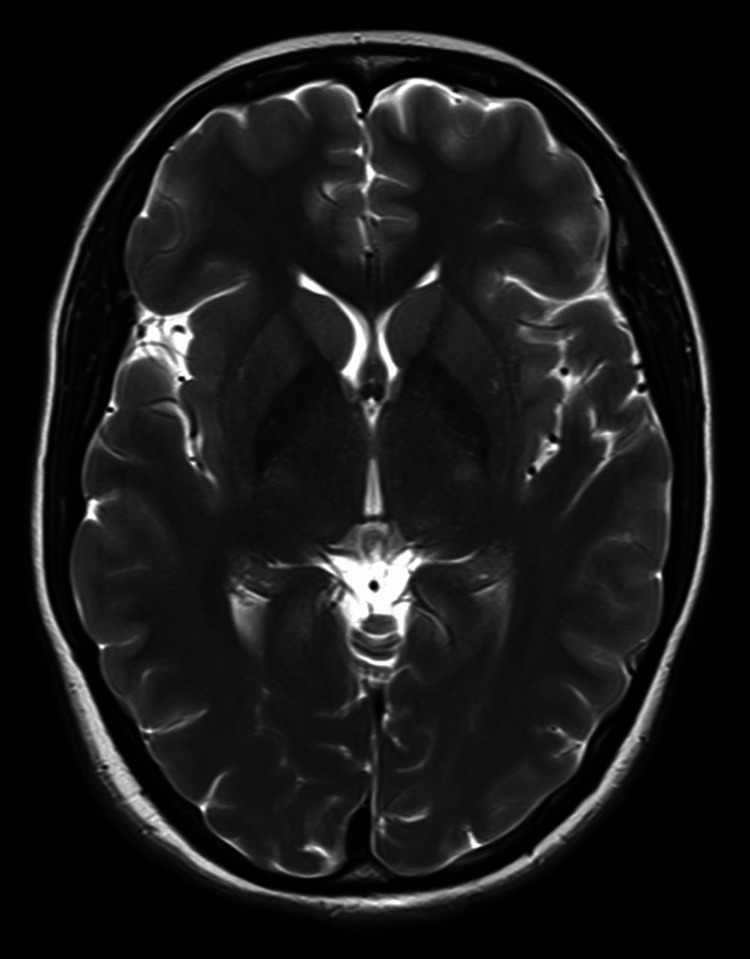
MRI brain with normal results

A 28-year-old female, P2L1, underwent an emergency LSCS for meconium-stained liquor under subarachnoid block using a low dose of 0.5% hyperbaric bupivacaine. About three hours postoperatively, she developed a reduced GCS with headache, followed by three episodes of GTCS, each lasting 2-3 minutes. Chest auscultation revealed bilateral coarse crepitations suggestive of aspiration. She was promptly intubated, shifted to the ICU, and managed on mechanical ventilation. An MRI of the brain was done, which came out to be normal (Figure 2). The patient was managed conservatively with anti-edema therapy and antiepileptics, and GCS improved subsequently. The patient was successfully extubated after four days of admission. The patient was monitored for an additional 48 hours for any neurological abnormalities or need for re-intubation. During this period, the patient remained asymptomatic and hemodynamically stable and was later discharged.

## Discussion

Intrathecal bupivacaine is widely used in obstetric anaesthesia because of its rapid onset, reliable sensory-motor blockade, and established efficacy for caesarean delivery [1-3]. However, our case series highlights an unusual pattern of bupivacaine-induced neurotoxicity occurring exclusively in pregnant patients, with all events linked to a single suspected faulty batch of hyperbaric bupivacaine. Although rare, similar severe neurological events, including seizures and status epilepticus, have been documented following intrathecal bupivacaine in non-pregnant adults [6,7], supporting the possibility of either batch-related contamination or increased susceptibility in selected patient groups. These cases highlight the importance of maintaining a high index of suspicion for neurological complications, despite the strong safety record of bupivacaine in obstetric anaesthesia.

The precise mechanisms underlying intrathecal bupivacaine neurotoxicity remain uncertain. Proposed explanations include direct neuronal injury within the spinal cord [8], disruption of neuronal membrane stability and ion channel function [9], and concentration-dependent excitotoxicity [8]. Experimental studies demonstrate a clear relationship between dose, concentration, and neurotoxic changes, underscoring the narrow therapeutic margin of bupivacaine when administered intrathecally [8,11].

Pregnancy-related physiological changes may further contribute to heightened vulnerability. Reduced cerebrospinal fluid volume, engorged epidural veins, and increased intra-abdominal pressure, well-documented in parturients, facilitate greater cephalad spread of intrathecal local anaesthetics [1-3]. These factors may increase central nervous system exposure even when standard doses are used. In addition, hormonal influences during pregnancy may modify neuronal excitability and responsiveness to local anaesthetics, potentially amplifying susceptibility to toxicity [4,5].

Although systemic local anaesthetic toxicity is a recognised cause of seizure activity, seizures following intrathecal bupivacaine are exceedingly rare. Existing reports describe severe neurological manifestations, including status epilepticus and transient neurological symptoms, after intrathecal bupivacaine administration in non-pregnant patients [6-10]. The temporal clustering of events in our series, combined with the identification of a shared lot number, raises concern for a defective batch. Potential mechanisms include altered potency, degradation of the active ingredient, contamination with neurotoxic impurities, or variation in baricity leading to inconsistent spinal spread [8-10].

A striking feature of this series is that only pregnant women were affected, whereas non-pregnant individuals who received the same preparation experienced no complications. This pattern suggests a pregnancy-specific vulnerability rather than uniformly increased toxicity caused by the batch itself. Prior studies have demonstrated significantly greater cephalad spread of intrathecal local anaesthetics in parturients compared with non-pregnant patients receiving identical doses [1-3], providing a plausible explanation for why only parturients exhibited neurotoxic manifestations.

## Conclusions

This case series suggests that pregnant patients may be uniquely vulnerable to neurotoxic effects from degraded or impure bupivacaine, owing to altered physiological states. Further research is needed to clarify mechanisms and ensure safe anaesthesia practices in obstetric care. Strengthening drug quality monitoring, maintaining vigilance for atypical neurological symptoms, and promptly reporting adverse events will be crucial in preventing similar events in the future. Collaborative research and larger surveillance studies are essential to better understand this rare but clinically significant complication.

## References

[1] Santos A, Pedersen H, Finster M, Edström H (1984). Hyperbaric bupivacaine for spinal anesthesia in cesarean section. Anesth Analg.

[2] Chung CJ, Bae SH, Chae KY, Chin YJ (1996). Spinal anaesthesia with 0.25% hyperbaric bupivacaine for caesarean section: effects of volume. Br J Anaesth.

[3] Roofthooft E, Van de Velde M (2008). Low-dose spinal anaesthesia for caesarean section to prevent spinal-induced hypotension. Curr Opin Anaesthesiol.

[4] Ferrarezi WP, Braga AF, Ferreira VB, Mendes SQ, Brandão MJ, Braga FS, Carvalho VH (2021). Spinal anesthesia for elective cesarean section. Bupivacaine associated with different doses of fentanyl: randomized clinical trial. Braz J Anesthesiol.

[5] Shafiei FT, Codd PJ (2023). Bupivacaine. StatPearls.

[6] Vanmarcke A, Lormans P, Vandewaeter C (2022). Status epilepticus following intrathecal administration of bupivacaine: a case report. J Investig Med High Impact Case Rep.

[7] Song W, Zhang H, Li X, Yu C, Zhou Y, Li Y, Chen B (2023). Delayed lethal central nervous system toxicity induced by a low-dose intrathecal administration of bupivacaine: case report. Front Anesthesiol.

[8] Takenami T, Yagishita S, Murase S, Hiruma H, Kawakami T, Hoka S (2005). Neurotoxicity of intrathecally administered bupivacaine involves the posterior roots/posterior white matter and is milder than lidocaine in rats. Reg Anesth Pain Med.

[9] Forget P, Borovac JA, Thackeray EM, Pace NL (2019). Transient neurological symptoms (TNS) following spinal anaesthesia with lidocaine versus other local anaesthetics in adult surgical patients: a network meta-analysis. Cochrane Database Syst Rev.

[10] Vyshka G, Vacchiano G (2014). Severe flaccid paraparesis following spinal anaesthesia: a sine materia occurrence. BMJ Case Rep.

[11] Pollock JE, Neal JM, Stephenson CA, Wiley CE (2003). Neurotoxicity of spinal local anesthetics and transient neurological symptoms. Reg Anesth Pain Med.

